# Association of the *ABCB1* gene polymorphism *C3435T* (rs1045642) with acute myeloid leukemia: A genetic study

**DOI:** 10.1016/j.htct.2025.106239

**Published:** 2026-01-10

**Authors:** Roh Ullah, Nazish Mazari, Ghulam Mustafa, Aisha Hameed, Shagufta Khaliq, Ali Amar, Faiz Ul Haq, Asif Haleem Khan, Asif Naveed

**Affiliations:** a Department of Haematology, University of Health Sciences, Lahore, Pakistan; b Department of Pathology, Isfandyar Bukhari DHQ Hospital Attock, Pakistan; c Department of Pathology, Gujranwala Medical Collage, Pakistan; d Department of human genetics and molecular biology, University of Health Sciences, Lahore, Pakistan; e Department of Microbiology, University of Health Sciences, Lahore, Pakistan

**Keywords:** *ABCB1*, *C3435T*, P-gp, AML, PCR-RFLP

## Abstract

**Introduction:**

The *ATP Binding Cassette Subfamily B1* (*ABCB1*) gene is responsible for encoding the permeability glycoprotein (P-gp), a crucial protein involved in multidrug resistance. P-gp functions as an ATP-dependent efflux pump, actively removing diverse substances, including carcinogens, from cells. However, a specific genetic variation called the *C3435T* polymorphism of the *ABCB1* gene has been linked to reduced plasma levels of P-gp substrates. This genetic variation leads to the accumulation of harmful compounds within cells, which may increase susceptibility to hematological malignancies. This study aims to determine the frequency of *ABCB1* gene polymorphism *C3435T* (rs1045642) in acute myeloid leukemia patients at tertiary care hospitals in Lahore, Pakistan.

**Methods:**

A cross-sectional comparative study was conducted to investigate the association between *ABCB1* gene polymorphism (*C3435T*) and acute myeloid leukemia. A total of 100 samples (50 cases and 50 healthy controls) were genotyped using restriction fragment length polymorphism assay.

**Results:**

The TT genotype of *ABCB1 C3435T* was more prevalent in cases (62%) compared to the control group (20%). In different genetic models, the TT genotype was significantly associated with acute myeloid leukemia when compared to the CC and CT genotypes.

**Conclusion:**

This study suggests that the TT genotype of the *ABCB1 C3435T* gene polymorphism is more strongly associated with acute myeloid leukemia compared to controls. This specific genotype may contribute to the development or progression of this malignancy. Further research is needed to explore the functional implications of this genetic variation in the pathogenesis.

## Introduction

Acute Myeloid Leukemia (AML) is a rapidly progressing blood cancer characterized by the uncontrolled growth of myeloid blast cells in the bone marrow and peripheral blood [1,2]. The development of AML can be influenced by various factors, including exposure to radiation, certain medications, chemicals, and genetic predisposition [3]. Among the genetic factors, polymorphisms in genes involved in xenobiotic and drug transporters have been implicated as potential risk factors for AML [4].

One such gene is the *ATP Binding Cassette Subfamily B1* (*ABCB1*), also known as the *Multidrug Resistance 1* (*MDR1*) gene, located on chromosome 7. The *ABCB1* gene is highly polymorphic, with over 50 reported single-nucleotide polymorphisms (SNPs) [4]. Among these SNPs, the *C3435T* polymorphism in exon 26 has been extensively studied [5].

The *ABCB1* gene encodes a protein called permeability glycoprotein (P-gp), which functions as an ATP-dependent efflux pump, actively transporting various substances, including carcinogens, out of the cells [6]. The *C3435T* polymorphism of the *ABCB1* gene has been associated with a significant reduction (more than two-fold) in plasma concentration of P-gp substrates [7]. Decreased expression of P-gp can lead to the accumulation of xenobiotics and toxic compounds within cells, potentially increasing the risk of hematological malignancies and other diseases [8].

Several studies have investigated the relationship between the *ABCB1* gene polymorphism, *C3435T*, and acute leukemia in different populations. Therefore, this study aims to determine the frequency of the *ABCB1 C3435T* gene polymorphism in AML patients at tertiary care hospitals in Lahore, Pakistan. By examining this association, we can gain further insights into the genetic factors contributing to AML susceptibility in this population.

## Material and methods

A cross-sectional comparative study was conducted involving 100 samples: 50 patients diagnosed with AML and 50 healthy controls. The samples were collected at Jinnah Hospital and Shaikh Zayed Hospital in Lahore, with the necessary approvals from ethics review committees. The research was carried out in the Hematology & Human Genetics and Molecular Biology departments at the University of Health Sciences in Lahore.

Informed consent was obtained from all AML patients who agreed to participate and 3 mL of blood were collected in ethylenediaminetetraacetic acid (EDTA) vacutainers from each. Detailed information was collected using a specially designed proforma. AML diagnosis was based on clinical presentation, peripheral and bone marrow morphology, cytochemistry, and immunophenotyping. Age- and sex-matched healthy individuals were enrolled as a control group.

Genomic DNA extraction from frozen EDTA blood samples was performed using the phenol-chloroform-isoamyl method. The extracted DNA was stored at −20 °C. The detection of the *ABCB1* (*C3435T*) gene polymorphism was performed using a Polymerase Chain Reaction-Restriction Fragment Length Polymorphism (PCR-RFLP) assay. Following genomic DNA extraction, the targeted region in exon 26 of the *ABCB1* gene was amplified by PCR.

Each PCR assay was performed in a final reaction volume of 15 µL with 2.5 µL genomic DNA, 8 µL universal master mix, 0.2 µL *ABCB1 C3435T* forward primer, 0.2 µL *ABCB1 C3435T* reverse primer, and 4.1 µL distilled water. A thermal cycler was used for the PCR process, which consisted of an initial denaturation step, followed by cycles of denaturation, annealing, and extension. The final extension was performed, and the amplified PCR product, sized at 244 base pairs (bp), was obtained.

**Figure 1:**
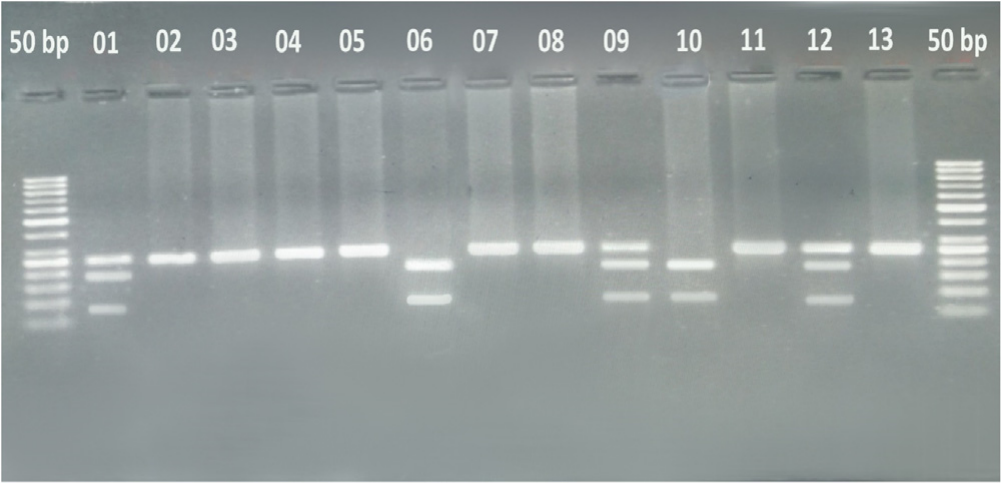
A representative gel photograph of the Polymerase Chain Reaction-Restriction Fragment Length Polymorphism assay. The gel depicts two DNA fragments in samples 6 and 10, indicating the CC genotype. Samples 1, 9, and 12 show three DNA fragments, representing the CT genotype. On the other hand, the remaining samples exhibit a single DNA fragment, indicating the TT genotype.

The Polymerase Chain Reaction (PCR) product was subsequently digested overnight with the MboI restriction enzyme at 37 °C to identify the polymorphism. The resulting fragments were then separated by 3 % agarose gel electrophoresis containing ethidium bromide and visualized under an ultraviolet transilluminator. A DNA molecular weight marker of 50 bp was used to determine the size of the PCR-RFLP products. The digestion with the MboI restriction enzyme allowed differentiation between wild and variant type alleles (homozygous and heterozygous genotypes). The CC genotype yielded two fragments of 72 and 172 bp, the TT genotype yielded one fragment of 244 bp, and the CT genotype resulted in three fragments of 72, 172, and 244 bp (Figure 1).

To confirm the genotypes observed in AML patient samples using the PCR-RFLP assay, further validation was performed through direct DNA sequencing. The 244 bp *ABCB1* gene sequence was analyzed using the online insilico software Nebcutter, which confirmed the number and sizes of the fragments after MboI digestion.

### Statistical analysis

Statistical analysis was conducted using SPSS version 24. The continuous variables such as age, hemoglobin level (Hb), red blood cell count (RBC), white blood cell count (WBC), platelet count, and blast percentage were reported as mean ± standard deviation (SD). Categorical variables including gender, AML subtypes, patient clinical features, and patient genotypes were presented as frequencies. The association between patient genotypes and AML was analyzed using the Chi-square test, and the odds ratio (OR) with a 95 % confidence interval (95 % CI) were calculated. A p-value <0.05 was considered statistically significant for all analyses.

## Results

In this study, 50 healthy control individuals, 38 (76 %) males and 12 (24 %) females, were examined. Among the 50 AML patients diagnosed, 40 (80 %) were male, and ten (20 %) were female. A marginal higher number of male patients was observed in the AML group. PCR-RFLP analysis of the *ABCB1* gene polymorphism (rs1045642) revealed the distribution of genotypes among the participants. Of the AML patients, 4 % had homozygous CC wild type alleles, 34 % had heterozygous CT alleles, and 62 % had homozygous TT alleles. The C and T allelic frequencies in the AML group were 21 % and 79 %, respectively. In the normal healthy control group, 16 % had homozygous CC wild type alleles, 64 % had heterozygous CT alleles, and 20 % had homozygous TT alleles. The C and T allelic frequencies in the control group were 48 % and 52 %, respectively.

Using the Chi-square test, a statistically significant difference was observed between the AML patients and the normal healthy controls (p-value ≤0.05). The frequency of homozygotes for the TT alleles in AML patients was significantly higher compared to normal healthy controls. Thus, the present study suggests a strong association between the homozygous TT genotype of the *C3435T* SNP and AML susceptibility, indicating that it may have a potential role in AML.

In the genotypic model, the TT homozygous genotype of the *C3435T* polymorphism was significantly associated with AML compared to the homozygous CC and heterozygous CT genotypes (OR = 16.2; 95 % CI: 0.38–12.70; p-value = 0.0001). In the dominant model, the combined CT and TT genotypes of the *C3435T* polymorphism showed an association with AML compared with the homozygous CC genotype (OR = 5.15; 95 % CI: 0.98–27.03; p-value = 0.032). Similarly, in the recessive model, the TT genotype of *C3435T* showed an association with AML when compared to the combined CC and CT genotypes (OR = 8.28; 95 % CI: 3.123–21.94; p-value = 0.0001).

By analyzing the genetic models, it was observed that the frequency of the TT genotype of the *ABCB1 C3435T* polymorphism was 16.24 times more prevalent than the CC genotype in the genotypic model. In the dominant model, the combined CT and TT genotypes of the *ABCB1 C3435T* polymorphism were 5.15 times more prevalent than the CC genotype. Furthermore, in the recessive model, the TT genotype of the *ABCB1 C3435T* polymorphism was 8.28 times more prevalent than the combined CC and CT genotypes. These findings suggest that the homozygous TT genotype of *ABCB1 C3435T* polymorphism is more associated with AML. The recessive model was identified as the best fit model for the data in the present study, which was further supported by the highly significant p-value of 0.0001 in the recessive model analysis (Table 1).

**Table 1:** Distribution of alleles and genotypes of the *ABCB1* (rs1045642) gene polymorphism and its association with AML.

*ABCB1* rs1045642	Frequency n (%)	
	AML cases (*n* = 50)	Healthy controls (*n* = 50)
**Genotype – n (%)**		
CC	2 (4)	8 (16)
CT	17 (34)	32 (64)
TT	31 (62)	10 (20)
**Allele – (%)**		
C	21	48
T	79	52
**OR statistics**	**OR (95% CI)**	**p-value**
CC versus TT (genotypic model)	16.24 (2.60–101.39)	0.0001
C versus T (allelic model)	3.47 (1.86–6.46)	0.0001
CC versus CT and TT (dominant model)	5.15 (0.98–27.03)	0.032
CC and CT versus TT (recessive model)	8.28 (3.13–21.94)	0.0001

AML: Acute Myeloid Leukemia; OR: odds ratio; 95 % CI: 95 % confidence interval.

To explore potential associations, the demographic, hematological, and clinical characteristics of AML patients were stratified based on the *ABCB1* gene polymorphism (*C3435T*). The combined CC and CT group was compared with the TT genotype group in respect to these characteristics. Overall, there were no statistically significant differences between the two genotype groups, except for lymphadenopathy, which showed clinical significance in the combined CC and CT genotype group (p-value = 0.015 - Table 2). This indicates that the presence of lymphadenopathy may have a different clinical implication in patients with the CC and CT genotypes compared to those with the TT genotype.

**Table 2:** Stratification of baseline characteristics of Acute Myeloid Leukemia patients by *ABCB1* (rs1045642) polymorphism.

Baseline Characteristics	*ABCB1* rs1045642 (Recessive model)		p-value
	CC and CT (*n* = 19)	TT (*n* = 31)	
**Demographic**			
Age (years)	39 ± 10.9	35 ± 10.2	0.820
Male - n ( %)	17 (89.5)	23 (74.2)	0.190
Female - n ( %)	2 (10.5)	25 (25.8)	0.282
**Hematological**			
Hemoglobin (g/dL)	8.5 ± 1.57	9.6 ± 1.48	0.470
Red Blood Cell Count (x 10^6^/µL)	3.55 ± 0.37	3.63 ± 0.38	0.953
White Blood Count (x 10^3^/ µL)	23.9 ± 7.5	22.1 ± 7.2	0.537
Platelet Count (x 10^3^/µL)	109 ± 28	108 ± 27	0.891
Blasts ( %)	65.4 ± 18.4	65.1 ± 19.2	0.821
**Clinical History** - n ( %)			
History of Fever	16 (84.2)	26 (83.9)	0.975
History of Bruises	9 (47.4)	12 (38.7)	0.547
History of Infection	11 (57.9)	21 (67.7)	0.481
Previous History of Hematological Malignancy	3 (15.8)	5 (16.1)	0.975
Family History of Cancer	4 (21.1)	10 (32.3)	0.392
History of Pallor	14 (73.7)	27 (87.1)	0.231
History of Gum Bleeding	3 (15.8)	6 (19.4)	0.750
History of Hepatomegaly	4 (21.1)	4 (12.9)	0.445
History of Splenomegaly	12 (63.2)	17 (54.8)	0.563
History of Lymphadenopathy	5 (26.3)	1 (3.2)	**0.015**

## Discussion

The main objective of this study was to identify the association between the *ABCB1* gene polymorphism (*C3435T*) and AML. Using PCR-RFLP for genotype distribution analysis, a significantly higher frequency of homozygous alleles (TT) was found in AML patients (62 %) when compared to the healthy control group (20 %; p-value ≤ 0.05). These results are in line with a study conducted by Li et al. in 2016, in whcih the authors also observed a significantly higher frequency of TT homozygous genotypes of the *ABCB1* gene polymorphism in AML patients (24.32 %) compared to healthy controls (15.56 %; p-value = 0.02) [9].

However, it is important to consider that a previous study focused on the Iraqi population and the *ABCB1* SNP (*C3435T*) reported insignificant differences in homozygous mutant-T allele frequencies between healthy individuals (55 %) and AML patients (56 %) [10]. These discrepancies could be attributed to variations in sample selection criteria, study design, sample size, and genetic diversity between different populations, which can influence the observed associations between the *ABCB1* gene polymorphism and AML susceptibility.

To enhance the reliability of the current findings and better comprehend the role of the *ABCB1* gene in AML development, further research with larger and more diverse populations is necessary.

In the present study, a significant association between the homozygous TT genotype and AML was also identified when compared to the homozygous CC and heterozygous CT genotypes. This aligns with findings from similar investigations by Feng et al., Ma et al., Li et al., and Meirav Kedmi et al. in various populations, all of which support the significant correlation between the *ABCB1 C3435T* polymorphism and AML [9,11, 12, 13].

However, it is noteworthy that some studies, including those by Jamroziak et al., Rao et al., and Kaltoum et al., reported opposing results, suggesting potential variations in the impact of the *ABCB1 C3435T* polymorphism on AML susceptibility across different populations [14, 15, 16]. Factors such as population-specific genetic backgrounds, environmental factors, and sample sizes may contribute to these inconsistencies.

In the current study, various genetic models were applied to assess the association between the *ABCB1 C3435T* polymorphism and AML. The findings revealed significant associations in co-dominant, dominant, and recessive models. Specifically, the TT homozygous genotype of *C3435T* was associated with AML compared to the homozygous CC and heterozygous CT genotypes, with consistent ORs across the models. These results corroborate a previous study conducted by Li et al., where the authors also reported an association between the *ABCB1 C3435T* polymorphism and AML using similar modeling approaches [9]. The consistency between the present study and previous research highlights the robustness of the association between the *ABCB1 C3435T* polymorphism and AML providing further evidence of its potential role in AML.

Two shortcomings of this study are the lack of an investigation of treatment outcomes and the effect of the *ABCB1 C3435T* polymorphism on the prognosis of AML. While this study focused on the genetic link between *ABCB1* polymorphism and AML susceptibility, future research should investigate its prognostic role to fully assess its relevance to patient outcomes.

## Conclusions

In conclusion, this study investigated the potential association between the *ABCB1* gene polymorphism (*C3435T*) and AML. A significant association was found between the homozygous TT genotype and AML, consistent with previous research. However, conflicting results from other studies suggest population-specific variations. Further research with larger and more diverse populations is needed to validate these findings. Understanding the role of the *ABCB1* gene in AML development can contribute to improved detection and personalized treatment approaches.

## Statements & declarations

**Funding:** The authors declare that no funds, grants, or other support were received during the preparation of this manuscript

## Author contributions

Authors Roh Ullah, Nazish Mazari, and Shagufta Khaliq contributed to the study conception and design. Material preparation, data collection and analysis were performed by Roh Ullah, Ghulam Mustafa, Asif Naveed, Aisha Hameed, Faiz Ul Haq and Ali Amar. The first draft of the manuscript was written by Roh Ullah, Faiz Ul Haq and Asif Haleem Khan and all authors commented on the versions of the manuscript. All authors read and approved the final manuscript.

## Ethics approval

The ethical committee of UHS Lahore Approved the study (erc/uhs/2018.008).

## Consent to participate

Informed consent was obtained from all individual participants included in the study

## Data availability

The data that support the findings of this study are available from the corresponding author upon reasonable request.

## Conflicts of interest

The authors declare no conflicts of interest.
